# Non-linear dimensionality reduction of signaling networks

**DOI:** 10.1186/1752-0509-1-27

**Published:** 2007-06-08

**Authors:** Sergii Ivakhno, J Douglas Armstrong

**Affiliations:** 1 Biological Engineering Division, Massachusetts Institute of Technology, Cambridge, MA 02139, USA; 2 School of Informatics, 5 Forrest Hill, University of Edinburgh, Edinburgh EH1 2QL, UK

## Abstract

**Background:**

Systems wide modeling and analysis of signaling networks is essential for understanding complex cellular behaviors, such as the biphasic responses to different combinations of cytokines and growth factors. For example, tumor necrosis factor (TNF) can act as a proapoptotic or prosurvival factor depending on its concentration, the current state of signaling network and the presence of other cytokines. To understand combinatorial regulation in such systems, new computational approaches are required that can take into account non-linear interactions in signaling networks and provide tools for clustering, visualization and predictive modeling.

**Results:**

Here we extended and applied an unsupervised non-linear dimensionality reduction approach, Isomap, to find clusters of similar treatment conditions in two cell signaling networks: (I) apoptosis signaling network in human epithelial cancer cells treated with different combinations of TNF, epidermal growth factor (EGF) and insulin and (II) combination of signal transduction pathways stimulated by 21 different ligands based on AfCS double ligand screen data. For the analysis of the apoptosis signaling network we used the Cytokine compendium dataset where activity and concentration of 19 intracellular signaling molecules were measured to characterise apoptotic response to TNF, EGF and insulin. By projecting the original 19-dimensional space of intracellular signals into a low-dimensional space, Isomap was able to reconstruct clusters corresponding to different cytokine treatments that were identified with graph-based clustering. In comparison, Principal Component Analysis (PCA) and Partial Least Squares – Discriminant analysis (PLS-DA) were unable to find biologically meaningful clusters. We also showed that by using Isomap components for supervised classification with k-nearest neighbor (k-NN) and quadratic discriminant analysis (QDA), apoptosis intensity can be predicted for different combinations of TNF, EGF and insulin. Prediction accuracy was highest when early activation time points in the apoptosis signaling network were used to predict apoptosis rates at later time points. Extended Isomap also outperformed PCA on the AfCS double ligand screen data. Isomap identified more functionally coherent clusters than PCA and captured more information in the first two-components. The Isomap projection performs slightly worse when more signaling networks are analyzed; suggesting that the mapping function between cues and responses becomes increasingly non-linear when large signaling pathways are considered.

**Conclusion:**

We developed and applied extended Isomap approach for the analysis of cell signaling networks. Potential biological applications of this method include characterization, visualization and clustering of different treatment conditions (i.e. low and high doses of TNF) in terms of changes in intracellular signaling they induce.

## Background

A major challenge for systems biology is to understand the combinatorial regulation in signaling cascades and transcription initiation mechanisms that underlie complex cellular behaviors. Signaling pathways regulate essential processes in living cells and determine cellular responses to external stimuli. Traditional biochemical and molecular biology approaches focus on functional contributions of individual molecules and often overlook that activation of multiple signaling molecules determines cellular response to external stimuli. Such information will be crucial for constructing predictive and descriptive models of cell decision processes and identifying how pathological conditions such as uncontrollable cellular proliferation arise from abnormalities in signaling networks. In contrast to transcriptional regulatory networks for which high-throughput technologies such as DNA microarrays, genome-wide knockouts/RNAi and chromatin-immunoprecipitation are available for profiling network activities, the analysis of signaling networks at the biochemical/molecular level has been hindered by the absence of sensitive high-throughput approaches. Recently, several such methodologies have been described that use either multiparameter flow cytometry with causal (Bayesian) networks [1,2] or combinations of protein signaling assays with Bayesian networks [3], Principal Components Analysis (PCA) [4], and Partial Least-Squares Regression (PLSR) [5].

**Figure 1:**
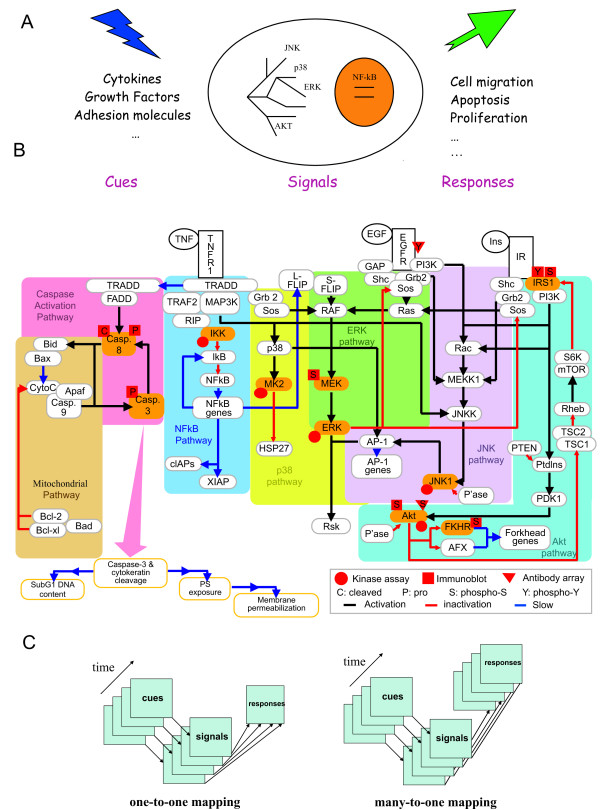
**Features of the Cytokine Compendium and the cues – signals – responses paradigm**. A. Cues – signals – responses paradigm for the design and execution of systems biology experiments for the analysis of signaling networks. Cells are exposed to perturbations (cues) and molecular signaling molecules and cellular responses are assayed, followed by application of multivariate statistical and machine learning data analysis techniques. B. Schematic representation of the apoptosis signaling network induced by TNF, EGF and insulin. On the diagram arrows indicate the type of interaction: activation (green), inhibition (red) and slow process (blue). The measured proteins (molecular signals) are highlighted in yellow. Red circles, triangles, and rectangles indicate kinase assay, antibody array, and western blotting measurements, respectively. The decomposition of the network into different pathways is shown by different colors (reproduced with modifications with permission from [5]). C. Distinction between one-to-one and many-to-one mapping functions between signals and responses. In the former case cellular response is measured for each time point of a particular treatment condition (cue), in the later – one cellular response is measured after several time points of the treatment condition.

The typical approach for studying cellular signaling in such experiments is based on the notion of cues – signals – responses paradigm and involves several steps in experimental design and computational modeling (Figure 1A) [6]. First, one or several signal transduction cascades are chosen depending on the questions that are addressed. For example, the apoptosis signaling network can be selected to study different rates of apoptosis in cancer cells. Since even a small number of signaling cascades may include hundreds of proteins and other signaling molecules, this step also involves selection of the smaller subset of signaling proteins, "signals", which are believed to be the most relevant for the regulation of a signaling network (based on the background biological knowledge and availability of appropriate high-throughput technology). As a final step in this design phase, the choice for specific perturbations – "cues" – is made to induce changes in the information flow through the signaling network and a number of specific cellular responses are assayed to analyze output of the network. For example, in the apoptosis signaling networks, different combinations of cytokines and growth factors can act as cues and assays measuring apoptosis intensity can act as responses.

In the second step the activity and concentration of signaling molecules as well as corresponding cellular responses are measured experimentally across different cues/treatment conditions. Possible experimental approaches include western blotting, high-throughput kinase activity assays and protein microarrays [7]. Assays for quantification of cellular responses vary depending on the specific application and may include measurements of cell migration, overall cell integrity or secretion of specific ligands.

The third and final step involves data analysis that addresses issues of building predictive and descriptive models of signaling networks. For instance, principal component analysis has been used to find how different cues and treatment conditions are positioned in the low-dimensional subspace of intracellular signals [4]. Alternatively, information on the activity and amount of intracellular signaling molecules was used to build PLSR model for prediction of cellular responses and selection of the most informative for classification subset of signals (feature selection) [5]. It should be noted that three steps of the systems biology methodology outlined above can be extended in various ways, for example the raise in activity of signaling molecules in response to different cues can be measured along many time points.

In this study we investigated the relationship between cues, signals and responses using two different cell signaling networks: apoptosis signaling network in human adenocarcinoma cells and the survey of multiple signaling pathways in 264.7 macrophages.

To analyze the apoptosis network we considered a previously published protein signaling dataset known as the Cytokine compendium [6], for which quantitative western blotting, high-throughput protein kinase assays and protein microarrays were used to investigate the combinatorial effect of tumor necrosis factor (TNF), epidermal growth factor (EGF) and insulin on apoptosis of human adenocarcinoma cells. The question addressed in the original study was how different combinations of three cytokines influence cancer cell death or survival. For example, depending on its concentration and the presence of other cytokines, TNF can have either a proapoptotic or prosurvival effect [8]. Consequently, the original aim was to investigate how the apoptosis signaling network was activated in response to different levels of TNF, EGF and insulin and how such differential activation contributes to antagonistic cellular decisions, such as cell death versus survival in cancer cells. By measuring activities of 19 different intracellular molecular signals (signaling molecules known to be associated with TNF, EGF, or insulin signaling) during a 24 hour time course and relating measured activities to apoptotic responses, a highly effective PLSR model for signals governing apoptosis was constructed [5] (Figure 1B). The trained model predicted with 90% accuracy the apoptotic responses from the test data with new experimental conditions (perturbations that partially blocked the apoptosis signaling pathway), and found major principle components of molecular signals that contribute most to correct predictions.

However, it may not always be possible to apply a fully-supervised dimensionality reduction approach to study how changes in the activity of signaling network influence cellular responses. For instance, Janes et al [5] used measurements of network activity across multiple early time points to predict apoptosis outcome at later time points, whereas in some applications it may be desirable to relate signals to responses at identical time points [5]. In the context of the variable and conflicting cellular responses to the various TNF, EGF, and insulin treatment conditions noted above it would be useful to understand how different treatments are positioned within the resulting space of intracellular molecular signals.

The second signaling network represented a large-scale survey of pathway interactions in response to 231 combinations of 21 different ligands (double ligand screen) carried out by the Alliance for Cellular Signaling (AfCS) in RAW 264.7 macrophages [9]. The phosphorylated states of 21 proteins were measured after 1, 3, 10, and 30 minutes of treatment with ligands two study interactions between different ligands in terms of intracellular signaling space of phosphoproteins. The ligands were selected to stimulate a diverse set of signaling pathways through Toll-like receptors (TLRs), G protein-coupled receptors (GPCRs), cytokine receptors and tyrosine kinase receptors [see Additional file 1], many of which are co-activated during physiological signaling events. Responses included measurements of secretion of 18 cytokines.

Here we assessed applicability of unsupervised dimensionality reduction techniques for the analysis of these two signaling networks. Our key aim was to infer connections between external cues, intracellular molecular signals and corresponding cellular responses. More specifically, by using Cytokine signaling data compendium and AfCS double ligand screen we aimed to answer the following questions:

1. Can different treatment combinations of ligands be positioned into separate clusters of cues in the low-dimensional signaling space?

2. What are characteristics of these clusters in the low-dimensional space (unsupervised learning)?

3. Can low-dimensional embedding be intuitively explained in terms of original dimensions of molecular signals?

4. Can the low-dimensional representation of signaling networks be used for predictive (supervised) modeling of cellular responses, i.e. apoptosis intensity?

5. Are there any differences in performance between linear and nonlinear dimensionality reduction techniques in the case of both supervised and unsupervised learning (i.e. PCA vs. Isomap)?

To address these questions we applied a non-linear dimensionality reduction approach Isomap [10]. Isomap and a similar technique, local linear embedding (LLE) [10,11] have already been successfully applied as dimensionality reduction approaches for gene networks [12-14] and many other problems in cognitive sciences and computer vision. These algorithms have been found superior to PCA and Multidimensional Scaling (MDS) in finding low-dimensional submanifolds in many cases. For instance, a modified LLE algorithm Local Context Finder (LCF) enabled successful reconstruction of a low-dimensional representation of the pathogen induced gene network in Arabidopsis [15].

**Figure 2:**
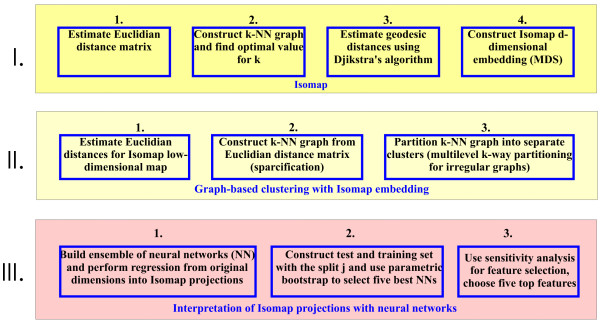
**Schematic representation of the extended Isomap approach**. Extended Isomap approach involves three main steps: Isomap algorithm to construct a low dimensional embedding of the apoptosis signaling networks (I), graph-based clustering to find clusters in the Isomap space (II) and neural networks ensemble to find meaningful interpretation of the Isomap projection (III)

To apply Isomap specifically in the context of signaling networks we extended it with graph-based clustering (questions 2) and neural networks (questions 3) (Figure 2). Finally, we used the low-dimensional embedding found by Isomap to build a classifier for prediction of apoptosis intensity (question 5). In our application Isomap generates a low-dimensional projection of signaling networks represented by the activity of molecular signals, where groups of different cues/treatment conditions could be easily identified and visualized (henceforce we refer to such projection as low-dimensional embedding of the signaling network). Consequently, the main contribution of this paper is analysis of signaling networks in the new unsupervised learning context using nonlinear dimensionality reduction approaches.

## Results

### Cytokine compendium dataset

The main details of the Cytokine compendium dataset [6] that are relevant for the present study are as follows. HT-29 epithelial cancer cells were treated with 10 combinations of saturating or subsaturating concentrations of TNF, EGF and insulin (0, 0.2, 5, 100 ng/ml TNF and 0, 1, 100 ng/ml EGF or 0, 1, 5, 500 ng/ml insulin respectively), which collectively represent all the cues used in the study. 19 molecular signals were chosen to characterize changes in signaling network activity in response to each cue condition [see Additional file 1]. Finally, to incorporate temporal information 13 measurements were made during the 24 hour time course after treatment with cues (5, 15, 30, 60, 90 min, 2, 4, 8, 12, 16, 20, 24 hr). Each measurement was performed in triplicate to assess reproducibility of individual assays. To correlate apoptosis level in HT-29 cells to measured signals in the network, cell-death phenotype was measured for each of 10 different cytokine combinations using four distinct apoptosis assays that characterized early (phosphatidylserine exposure), middle (caspase substrate cleavage and membrane permeability), and late (nuclear fragmentation) apoptotic responses.

#### Data representation and transformation

In our analysis of the Cytokine compendium each measurement comprises a single treatment condition at a distinct time point (e.g. TNF 100 ng treatment at 5 min). This representation gives 130 data points in 19 dimensions of molecular signals and produces a one-to-one mapping between the apoptosis signaling network and associated cellular responses. Such data prepossessing is different from the one used by Janes et al [5] where the whole time course for 19 molecular signals and additionally derived metrics were used to predict apoptosis for the 10 distinct combinations of cytokine treatments (many-to-one mapping) (Figure 1C). The approach of Janes et al leads to the PLRS regression model with only ten data points in 200-dimensional space, which for leave-one-out cross-validation (LOOCV) may in some instances lead to the problem of high variance. Considering each time point as an individual measurement here allows us to avoid this problem. In addition, it allows us to investigate the activity of the apoptotic signaling network in more detail, in particular how early and later time points correlate under different cytokine treatment conditions.

### PCA and PLS-DA could not find low dimensional embedding of apoptosis signaling networks

**Figure 3:**
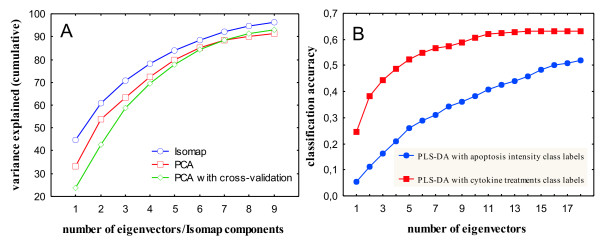
**Performance comparison of Isomap, PCA and PLS Discriminant Analysis on the Cytokine compendium dataset**. Panel A shows cumulative residual variance after the application of Isomap and PCA on the Cytokine compendium dataset. Panel B shows classification accuracy of PLS Discriminant Analysis with two different choices of class labels.

First we applied PCA to the Cytokine compendium to investigate if this linear dimensionality reduction technique can find meaningful clusters in a low-dimensional representation of the apoptosis signaling network. PCA was unable to find an optimal embedding: the first two leading eigenvalues accounted for just 53% of the variance (Figure 3A) and the reconstructed two-dimensional map had broad and unclear patterns and clusters [see Additional file 1]. Similar results were obtained when alternative cross-validation technique was applied to find PCA components (Figure 3A). PCA selects components that best explain variance in the data, and they may not be the ones with the strongest predictive power for apoptosis intensity or/and TNF-EGF-Insulin treatment conditions. To test if incorporation of class labels information improves separation of cytokine treatments we used PLS Discriminant Analysis (PLS-DA), which maximizes separation between groups of observation/response variables by rotating PCA components such that the maximum separation among different classes is obtained. Two PLS-DA experiments were performed on the Cytokine compendium. In the first, apoptosis intensity was discretized to represent class labels for low, medium and high rates of apoptosis; in the second, class labels were the 10 different cytokine treatment combinations. With this approach we were able to test assumptions of linearity both from treatment conditions to intracellular signaling space and from intracellular signaling space to apoptosis responses. Classification accuracy obtained via 10 fold cross validation for PLS-DA + cytokine treatments class labels and PLS-DA + apoptosis intensity class labels were 0.17 and 0.445 respectively for the first three principal components (Figure 3B). These results suggest that both supervised and unsupervised linear dimensionality reduction may ineffectively characterize the apoptosis signaling network when attempting the one-to-one signals-to-responses mapping.

We sought to apply our extended Isomap approach to see if non-linear dimensionality reduction can find the low-dimensional embedding of the apoptosis signaling network. Extended Isomap approach involves three main steps: The Isomap algorithm first constructs a low-dimensional embedding of the apoptosis signaling network, graph-based clustering with multilevel k-way partitioning then finds clusters in this low-dimensional space and an ensemble of neural networks are used to find meaningful interpretation of Isomap components in terms of original dimensions.

We have extended Isomap with graph-based clustering specifically to analyze noisy biological data. The original algorithm and most of its later applications dealt with high dimensional image and text data, e.g. images of the same object, shifted by small angles [11], where a low-dimensional continuous submanifold can be found. Consequently, Isomap projection is often used in machine vision and text mining for visualization and dimensionality reduction purposes. Such continuity of submanifold is not an intrinsic feature of signaling networks in cells treated with different cytokines or growth factors, which often activate distinct (but overlapping) subsets of proteins. In systems biology applications the detection of functional subsets within biological networks is of primary importance, since this allows to find clusters in the signaling space that correspond to similar cytokine treatment conditions.

**Figure 4:**
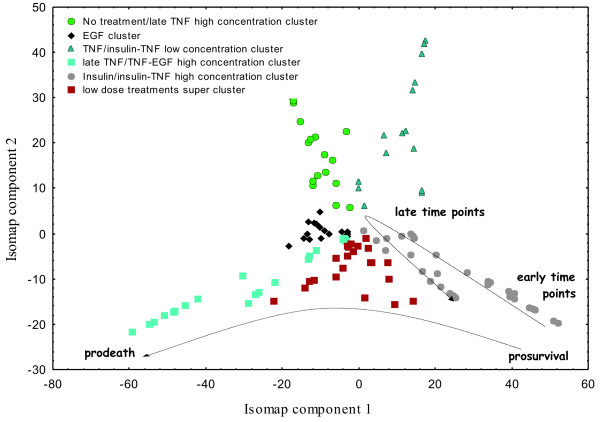
**Two dimensional Isomap projection of the Cytokine compendium dataset**. Clusters were identified using graph-based clustering with 20-nearest neighbours distance matrix and multilevel k-way partitioning scheme for irregular graphs. High concentration Isnulin/Insulin-TNF, EGF and TNF/TNF-EGF clusters are located in the lower part of the map; the general change of the apoptosis network activity from prosurvival to prodeath is shown by the line. High concentration Insulin and insulin-TNF clusters show temporal evolution of the apoptosis network activity from early to late time points.

**Figure 5:**
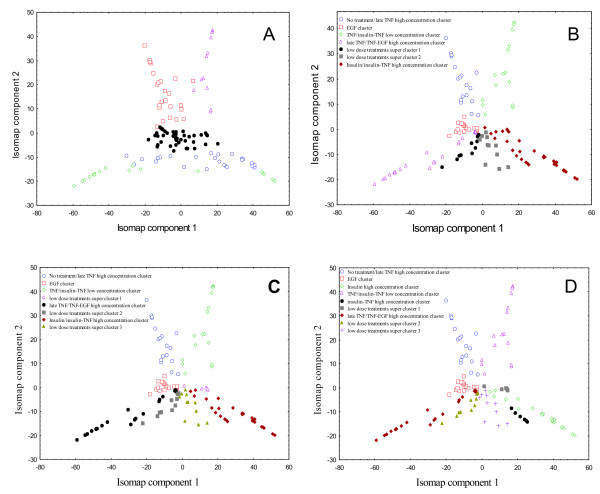
**Analysis of the Isomap projection of the Cytokine compendium dataset**. On the Panel A the cytokine compendium dataset was projected into the low-dimensional subspace using Isomap. Next, clusters were identified using the Expectation Maximisation (EM) clustering algorithm with the number of clusters set to 6 (clusters are labelled with different symbols). Panels B-C: Graph-based clustering of the two-dimensional Isomap embedding using the same parameters as for graph-based clustering in Figure 4, but with the expected number of clusters increased from 7 to 9, original clusters remain intact and partitioning is made across the super cluster. When the number of clusters is 9 the Insulin/Insulin-TNF high concentration cluster is partitioned in separate Insulin and Insulin-TNF clusters.

After applying Isomap we found that the first three Isomap components captured 71% of the variance (62% for the first two components), suggesting that Isomap was able to find a low-dimensional embedding of the apoptosis signaling network. By using graph-based clustering in the first two Isomap components we identified distinct clusters corresponding to different cytokine treatments (Figure 4). Closer evaluation of the five recovered clusters indicates that all of them convey information about original cytokine treatments. In particular, Isomap found clusters that reflect high concentration of insulin-TNF, mixed insulin-TNF and EGF-TNF high/medium treatment conditions. Moreover, the first Isomap component largely explains changes in the apoptosis network activity from prosurvival to prodeath. One additional noteworthy observation is the non-globular shape of most clusters found by graph-based clustering (k-nearest neighbors distance). Expectation Maximization based clustering that uses Euclidian distance and tends to detect globular clusters failed to identify clusters that reflect biologically meaningful treatment conditions (Figure 5A). This suggests that (I) neighborhood properties for a particular treatment condition captured by k-nearest neighbors distance are important for identification of clusters in the Isomap space and (II) the apoptosis signaling network tends to span low-dimensional embedding in a thread-like fashion rather than forming globular subclusters. Since in our case the network is defined by the different cytokine treatments (combination of EGF, TNF and Insulin) this indicates that changes in cytokine combination and/or time at which the intracellular signals are measured leads to the directional changes in the apoptosis network activity, such as changes from insulin to insulin-TNF cluster.

### Features of cytokine clusters found by Isomap

Isomap was able to reconstruct not only the low-dimensional embedding of insulin-induced apoptosis network, but also captured temporal profiles of network activity, with early time points occupying far end of the map and later time points approaching the centre (Figure 4). The insulin cluster was closely followed by the insulin-TNF high concentration cluster on the map (Figure 4). This observation is consistent with current biological understanding of the role of insulin in the apoptosis signaling network, where insulin augments AKT (protein kinase B) activity. Insulin-TNF treatment leads to a rapid increase in AKT activity during the early time points and sustaines activation for 24 h, whereas with TNF alone the AKT response is much smaller in magnitude [6]. For insulin-TNF treatment, the AKT activity was held high for the first 12 hours and contributed to the decrease in the apoptosis rate. This indicates that insulin-TNF and insulin-alone treatments, which are most similar by their activity profiles in the Cytokine compendium, were successfully placed by Isomap in similar location on the low-dimensional embedding. Since AKT is considered to be responsible for a significant portion of prosurvival signaling in this apoptosis network, we hypothesized that Isomap grouped time courses with insulin-TNF and insulin alone due to preferential low-dimensional projection along AKT dimension. To test this hypothesis we applied Isomap after removing the AKT signal from the dataset. The distinct insulin-TNF and insulin-alone clusters disappeared from the two-dimensional map while other clusters were preserved confirming importance of the AKT signal for low-dimensional projection.

The late TNF/TNF-EGF high concentration cluster was reflected along horizontal axes to the insulin-TNF/insulin cluster, occupying the lower left corner on the map. Isomap was able to clearly separate these distinct treatments that operate through different (although overlapping) pathways in apoptosis. By separating TNF and insulin clusters, Isomap appropriately found differences in the signaling network in conjunction with differences in apoptotic cellular responses. Interestingly, Isomap also separated TNF-EGF and insulin-TNF time points on the two-dimensional map, revealing distinct antagonistic effects of EGF and Insulin on TNF-induced apoptosis pathway. As was recently suggested, EGF in combination with TNF directly antagonizes stress pathway signaling comprising early JNK1 activity, early MK2 activity, and late cleaved caspase-8. In contrast, insulin in combination with TNF both antagonizes stress signaling and induces separate prosurvival signaling pathways through phosphorylated AKT, insulin receptor substrate 1 (IRS1), Forkhead transcription factor (P-FKHR), and procaspase-3 metrics [5].

Another clearly identifiable cluster on the two dimensional map was the EGF-alone treatment cluster, located in the centre of the map. This cluster was more compact than the two previously described clusters, which reflects higher homogeneity between time points after EGF treatment. This particularly applies to middle – later time points after disappearance of the early transient peaks of AKT, MEK and ERK activities. We have found that one of the closest subclusters to the no treatment cluster was the late high concentration TNF cluster (Figure 4). This may be caused by the return of measured kinase activities to their basal state, primarily due to the onset of apoptosis following TNF treatment. However, further experiments are required for better understanding of these results.

Although Isomap found clusters in the network corresponding to treatments with high doses of cytokines/growth factors, it failed to identify most of the clusters treated with low doses, which formed one supercluster of several low dose treatment conditions. For instance, low doses of TNF-EGF and insulin-TNF clusters were almost inseparable on the Cytokine map. This indicates that the network was not perturbed sufficiently enough to show clearly distinct response phenotypes. The only two distinct clusters corresponding to low concentration treatments are the low TNF/insulin-TNF low concentration cluster and no treatment cluster. Interestingly, when the number of expected clusters in the graph-based clustering is increased from 6 to 9, all original clusters remain intact and partitioning is made across the super cluster (Figure 5B–D). However, even by increasing the number of clusters we were unable to further separate the low dose treatments super cluster. This might suggest that low concentration treatment conditions perturb apoptosis network less significantly than treatments with higher doses.

### Interpretation of components recovered by Isomap

An important characteristic of linear dimensionality reduction methods such as PCA is the intuitive interpretation of components in the low-dimensional space in terms of the original dimensions. Unfortunately, since exact one-to-one correspondence between the original and low-dimensional subspace can not be recovered through application of Isomap, it is difficult to interpret low-dimensional projections in terms of original multidimensional input space. However, in contrast to image data, input variables in the signaling space have direct biological interpretation (e.g. MAK kinase activity) and it is essential that such information is captured by low-dimensional embedding. We applied neural networks (NN) to approximate the nonlinear projection from original input space into low-dimensional space found by Isomap, followed by the sensitivity analysis to choose the best subset of predictive variables in the input space.

The subset of variables with the highest rank/scores (contributing most to the low-dimensional Isomap space) includes phosphorylation/activity of MAPK-activated protein kinase 2 (MK2, mean rank 4), insulin receptor substrate 1 (IRS1, mean rank 2), MEK kinase (mean rank 3), AKT kinase (mean rank 1), and procaspase-3 (ProC3, mean rank 5) level [see Additional file 1], which conform to the pervious study of [5]. However, in our case variables define Isomap projection through a nonlinear combination, the exact form of which remains unidentifiable. Interestingly, they are not the obvious ones that would have been chosen based on our current understanding of the regulation of apoptosis (i.e. caspases). The presence of proteins from different kinase pathways (p38, ERK and ANT) as significance variables further suggested that only systems-level view can accurately model mapping functions in signaling networks. Another noteworthy feature is that Isomap components include not only caspases as immediate downstream effectors of apoptosis, but also more upstream signals such as insulin receptor substrate 1.

### Supervised comparison of PCA and Isomap

Many non-parametric classification algorithms in machine learning such as k-nearest neighbors (k-NN) perform poorly in a multidimensional space where pairwise distances between input datapoints become large. Consequently, we sought to compare classification accuracy of several machine learning algorithm using apoptosis intensity as class labels and original 19-dimesnional space of molecular signals, Isomap components space and PCA components space as input datasets. We assigned class labels representing 3 levels of apoptosis intensity (low, medium and high) to each time point using expectation maximization clustering and performed classification with support vector machines (SVM), k-NN or quadratic discriminant analysis (QDA) in three dimensional Isomap/PCA subspace and original multidimensional space (Table 1). K-NN and QDA classifiers based on Isomap dimensions showed comparable performance to classifiers that used original dataset and in both cases were better than PCA-based classifiers (ANOVA, p < 0.05). SVM did not detect statistically significant differences between PCA and Isomap dimensions. This in part can be due to more spherical clusters generated by PCA, which makes it easier for SVM to find the optimal margin in a low-dimensional space. Since the exact assignment of apoptosis signatures to each data point was infeasible due to the absence of experimental measurements, the accuracy of classification based on Isomap projection and original dataset was lower than that reported by Janes et al. using PLSR [5]. The difference in performance can most likely be attributed to different structure of mapping functions between cues and responses for the apoptosis signaling network (one-to-one vs. many-to-one) and is considered in detail below (Factors contributing to Isomap algorithm performance). We then tested whether Isomap projection could achieve higher accuracy on predicting late time points of apoptosis network activity using training data derived from early signals. K-nearest neighbors, trained on early time points to predict later time points showed much higher accuracy than when the training set was chosen randomly. In particular, a test error of 0.1 was obtained when the last three time points were used for testing (lower than when using full dataset - 0.22). This is consistent with previous reports [6] and suggests that protein activities at early times encode much of the information needed to specify an apoptosis-survival cell fate decision. It also indicates that Isomap could be used in predictive modeling and for determining long term behaviors in signaling networks.

**Table 1:** Error rates for SVM, k-NN and QDA used to compare effectiveness of PCA and Isomap for supervised classification analysis of the cytokine compendium (computed by 10-fold cross validation with 95% confidence intervals)

**Datasets**	**Isomap**	**Full data**	**PCA**
k-NN	0,31 ± 0.08	0,29 ± 0.08	0,39 ± 0.10
QDA	0,28 ± 0.07	0,23 ± 0.09	0,39 ± 0.08
SVM	0,31 ± 0.09	0,24 ± 0.08	0,36 ± 0.11

As the Cytokine compendium comprises time series data for each combination of cytokine treatments, it was important to confirm that the low-dimensional embedding recovered by Isomap is not solely governed by correlation between consecutive time points for each treatment condition. Consequently we introduced a modified Euclidian distance where points belonging to the same treatments receive more weight than points belonging to different treatments to see how this affects continuity of the submanifold found by Isomap. The distance D' is defined by the formula:

D'(xi,xj)={ed(xi,xj)βyi≠yj1−e−d(xi,xj)βyi=yj
 MathType@MTEF@5@5@+=feaafiart1ev1aaatCvAUfKttLearuWrP9MDH5MBPbIqV92AaeXatLxBI9gBaebbnrfifHhDYfgasaacH8akY=wiFfYdH8Gipec8Eeeu0xXdbba9frFj0=OqFfea0dXdd9vqai=hGuQ8kuc9pgc9s8qqaq=dirpe0xb9q8qiLsFr0=vr0=vr0dc8meaabaqaciaacaGaaeqabaqabeGadaaakeaacqqGebardaahaaWcbeqaaiabbEcaNaaakiabcIcaOiabbIha4naaBaaaleaacqqGPbqAaeqaaOGaeiilaWIaeeiEaG3aaSbaaSqaaiabbQgaQbqabaGccqGGPaqkcqGH9aqpdaGabeqaauaabaqaceaaaeaadaGcaaqaaiabdwgaLnaaCaaaleqabaWaaSaaaeaacqWGKbazcqGGOaakcqWG4baEdaWgaaadbaGaemyAaKgabeaaliabcYcaSiabdIha4naaBaaameaacqWGQbGAaeqaaSGaeiykaKcabaacciGae8NSdigaaaaaaeqaaOGaemyEaK3aaSbaaSqaaiabdMgaPbqabaGccqGHGjsUcqWG5bqEdaWgaaWcbaGaemOAaOgabeaaaOqaamaakaaabaGaeGymaeJaeyOeI0Iaemyzau2aaWbaaSqabeaadaWcaaqaaiabgkHiTiabdsgaKjabcIcaOiabdIha4naaBaaameaacqWGPbqAaeqaaSGaeiilaWIaemiEaG3aaSbaaWqaaiabdQgaQbqabaWccqGGPaqkaeaacqWFYoGyaaaaaaqabaGccqWG5bqEdaWgaaWcbaGaemyAaKgabeaakiabg2da9iabdMha5naaBaaaleaacqWGQbGAaeqaaaaaaOGaay5Eaaaaaa@65F1@

where x_i _is an input vector and y_i _are corresponding labels for each of 10 treatment conditions, d(x_i_,x_j_) denotes Euclidian distance and β is a scaling factor defined as an average distance between all pairs of data points. Consequently, the dissimilarity between two points is equal to or greater than 1 if the cytokine treatments are different and is less than 1 if treatments are the same. To prevent overfitting, the parameter α = 0.65 is used to reduce the restriction that intra-class dissimilarity is less than 1. Using Isomap in combination with this distance metric we found that the low-dimensional embedding was distorted by disconnected components and 6 that were very distant from all clusters. This suggests that clusters found by Isomap were not solely grouped by correlation between similar time points for any particular treatment condition.

### AfCS double ligand screen dataset

#### Data representation and transformation

The data for AfCS double ligand screen of phosphoproteins was downloaded from Signaling Gateway website at [16] [see Additional file 1]. In the screen the phosphorylated states of the following proteins were recognized (site of phosphorylation in parenthesis): Akt (S473), ERK (T202/Y204), Ezrin/radixin/moesin (T567/T564/T558), GSK 3 α/β (S21/9), JNK (T183/Y185), p38 MAPK (T180/Y182), p40 phox (T154), ribosomal S6 (S235/236), NF-κB p65 (S536), PKC δ/θ (S643/676), PKCμ (S916), p90 RSK (S380), Smad2 (S465/467), STAT 1 (Y701), STAT 3 (Y705), and STAT 5 (Y694). Measurements were made after 1, 3, 10, and 30 minutes of treatment with ligands; basal phosphorylation was assessed in untreated cells. Cells were treated with each individual ligand and simultaneously with the two ligand combination. To correct for lane-to-lane variation in total protein loaded, in the downloaded dataset all signal intensities were already normalized against the signal for Rho-GDI detected by a phosphorylation insensitive antibody. After preliminary exploratory analysis we decided not to use the corresponding measurements of cytokines also available through the AfCS double ligand screen. In contrast to the Cytokine compendium data, the AfCS has not made effort to insure that the same culture plates are used for the assessment of both phosphorylation and cytokine secretion, and in fact concentration of some ligands was different for the two screens. Under such conditions reliable mapping between signals and responses can not be achieved, consequently we applied only Isomap unsupervised learning procedure to the AfCS double ligand screen data.

In our analysis of the AfCS double ligand screen each measurement represents a single treatment condition with a particular ligand at one time point. After removing observation in which more than 3 measurements of phosphoproteins were missing the dataset comprised 1006 data points in 21 dimensions of molecular signals. The final pre-processing included standardization of the dataset to the unit variance. For examples of scripts and software used in pre-processing see Additional Files 2, 3, 4, 5.

### Extended Isomap analysis of the AfCS dataset

**Figure 6:**
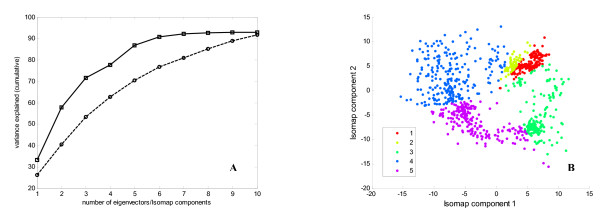
**Performance comparison of Isomap, PCA and PLS Discriminant Analysis AfCS double ligand screen dataset**. Panel A shows cumulative residual variance after the application of Isomap and PCA on the AfCS double ligand screen dataset. Panel B shows two dimensional Isomap projection of the AfCS double ligand screen dataset. Clusters were identified using graph-based clustering with 20-nearest neighbours distance matrix and multilevel k-way partitioning scheme for irregular graphs. Cluster 1 is functionally enriched for interferons, cluster 3 – for TLR ligands and cluster 5 for 2MA, PAF and UDP. Functional coherence scores for all clusters are listed in Additional file 1.

To determine the relationship between ligands in terms of changes in the phosphorylation levels of phosphoproteins they induce, and to find out whereas the multidimensional space of phosphoproteins can form the low dimensional embedding of the signaling network, we used Extended Isomap to analyze the AfCS double ligand screen data. The two dimensional map of the double ligand screen obtained after application of Isomap featured tight clusters and the first two dimensions explained 59% of the variance (Figure 6). In contrast, the corresponding two-dimensional PCA map gave unclear clusters [see Additional file 1] and the variance captured by the leading principal components was much lower – 40% (Figure 6B). The graph based clustering using the first two Isomap components identified 5 distinct clusters of different ligands treatment. To assess the biological significance of the clustering we calculated the functional coherence of clusters as the relative frequency (percentage) of occurrence of each ligand in each cluster normalized to the size of the cluster. Isomap clusters appeared to be functionally coherent [see Additional file 1]. For instance, cluster 3 on the Isomap map was significantly enriched for 3 out of 4 Toll-like receptor (TLR) ligands including lipopolysaccharide (LPS), P2C and P3C. Their functional coherence scores were 35%, 36% and 31% respectively. The emergence of these ligands as a single cluster is in agreement with similar patterns of protein phosphorylation they induce. TLR ligands signal through the proximal adaptor protein MyD88, which results in activation of NF-κB and MAP kinase pathways [17]. Interferons (IFNα, IFNβ and IFNγ) and interleukin 6 (IL6) were also grouped into a single cluster 1 (functional coherence scores 31%, 36%, 37% and 22% respectively) by their pattern of STAT phosphorylation [18]. 2-methylthio-ATP (2MA), platelet activating factor (PAF) and uridine diphosphate (UDP) also clustered together (functional coherence scores 38%, 32% and 30%), which is in agreement with similar phosphoprotein responses they induce [19]. On the contrary to Isomap, PCA has not found functionally coherent clusters [see Additional file 1] for TLRs ligands and interferons: interferons were split between clusters 2 and 4 and TLRs ligands – between clusters 1, 2 and 4 on the PCA two-dimensional map. Quite interestingly, after applying neural networks to determine the significance of individual variables we identified STAT 3/5 (mean rank 2 and 7) and JNK/P38 kinases (mean rank 1, 3 and 6) as the most highly represented phosphorproteins in the first two Isomap components [see Additional file 1]. The first two principal components were more homogeneous and included contribution of many different phosphorproteins at a similar level. In general, broad and undefined clusters on the PCA map are most likely attributed to the low informativeness of the leading two principal components that account only for the 40% of the variance. On the other hand, Isomap captures more information in the first two components and can therefore reconstruct the state of the signaling network more accurately.

## Discussion

### Extended Isomap can be used for visualization, predictive and descriptive modeling of apoptosis signaling network

If we consider signaling networks as multivariate nonlinear functions that map various perturbations and extracellular cues onto different cellular responses it becomes clear that only by understanding the network dynamics and interplay between its key components as a whole will we be able to construct predictive and descriptive models of cell decision processes. To explain different and sometimes antagonistic cellular responses to the same ligand, for example, TNF during angiogenesis [20], accurate measurements with frequent temporal sampling will be required. Since cells *in vivo *typically respond to multiple ligands all present at different concentrations, another important goal in signaling network research will be to identify how cells integrate and respond to multiple signals simultaneously. In this study we applied an unsupervised non-linear dimensionality reduction approach, extended Isomap, to find similar instantiations of apoptosis signaling network in response to different treatments with TNF, EGF or insulin. By projecting the original 19 dimensional space of intracellular signals into a 3-dimensional space, Isomap was able to reconstruct clusters corresponding to different cytokine treatments that were then identified with graph-based clustering. Alternative approaches were either unable to find biologically meaningful clusters (PLS-DA) or produced broad and unclear clusters (PCA). Potential biological applications of this method therefore include characterisation, visualisation and clustering of different treatment conditions (i.e. with low and high doses of TNF) in terms of the changes in intracellular signaling they induce. We also applied neural networks to infer the contribution that original variables make into the low-dimensional subspace. The variables contributing most to the low-dimensional projection were phosphorylation/activity of MEK, AKT, MK2, IRS1 and ProC3 cleavage, which is consistent with the previous reports [5]. Consequently, these signals might be potential targets for further biological investigation on how different doses and treatment conditions with TNF, EGF or insulin lead to different death/survival decisions in cancer cells.

Previous studies have concentrated on the structural reconstruction of the signaling networks using Bayesian networks [3] or their extension to learn causal (Bayesian) networks [1,2]. Other methods attempt to build signaling network regressors/classifiers that map cues into cellular responses, for example to build predictive models of migration speed in mouse fibroblasts [21]. Our extended Isomap approach instead focuses on a broader combination of unsupervised and supervised machine learning analysis of signaling networks through non-linear dimensionality reduction [11]. In this framework, different instantiations of the signaling networks (i.e. activities of signaling molecular induced by different growth factors) are first projected into low-dimensional space and then used to compare cues/treatment conditions by finding their corresponding low-dimensional embedding. Moreover, the relative contribution of each molecular signal to the projection can also be inferred. For instance, we showed that AKT activity is responsible for the segregation of the insulin cluster on the two-dimensional map. Although Isomap is primarily a technique for unsupervised learning and visualization, by feeding Isomap output into supervised classification algorithms such as k-NN and QDA, the technique can be successfully extended for predicting apoptosis outcomes. The Isomap predictive power was especially pronounced when early activation time points in the apoptosis signaling network were used to predict the later ones.

Extended Isomap also outperformed PCA on the AfCS double ligand screen data. Isomap identified more functionally coherent clusters than PCA and captured more information in the first two-components. We have not attempted to functionally characterize the AfCS double ligand screen as extensively as we analyzed the Cytokine compendium due to the large number of ligands and signal transduction pathways involved. Instead, our aim was to assess Isomap performance on much larger survey of signaling pathways and compare it to PCA. The Isomap projection becomes slightly worse when more signaling networks are analyzed (59% of the variance explained by the first two Isomap components for AfCS dataset versus 62% for the Cytokine compendium). For the PCA this reduction is much more dramatic – 53% and 40% respectively, suggesting that the mapping function between cues and responses becomes more non-linear when large signaling pathways are considered.

### Factors contributing to Isomap algorithm performance

Multiple factors could contribute to the superior performance of Isomap over linear dimensionality reduction approaches such as PCA and PLS-DA. First, the apoptosis signaling network in our representation may itself be nonlinear, especially when signaling molecules are located proximally to the receptors. Second, the application of Isomap for analysis of the apoptosis signaling network is similar to the notion of invariance manifolds in the field of cognitive neuroscience and in computer vision. In the invariance manifold, relevant features of the multi-dimensional input space are often represented by a large number of different instances of the same object to form a nonlinear surface in the low-dimensional output space [22]. This is the case, for example, with images of the same objects shifted by small angles (rotational invariance). Isomap has been successfully applied to many image processing problems having invariance manifold representation. Better performance of Extended Isomap approach for the analysis of signaling network might therefore be attributed to the fact that only a small portion of molecular signals, such as kinase activities, change significantly between different treatment conditions, especially when these conditions are similar (i.e. TNF-Insulin and Insulin alone treatment). Can activation profiles of other signaling networks be approximated by invariance manifolds? The invariance manifold concept suggests, in part, that as input cues are gradually varied (by changing concentration and/or composition of cytokines/growth factors), the activation state of intracellular signaling network also changes gradually. This systems-wide network view suggested by the non-linear dimensionality reduction differs from results obtained when studying smaller networks at the single cell level, where switch like behavior and population heterogeneity are documented (Albeck et al., Mathematical modeling and cell analysis of a snap-action feed-forward switch controlling receptor-mediated cell death, submitted). However, clusters found by Isomap do not perfectly reproduce invariance manifold, suggesting that further research into signaling networks dynamics at the systems wide level will be required to corroborate this idea.

When assessing performance of predictive models for signaling networks it is important to distinguish between afferent and efferent cascades from cues to intracellular signals and from intracellular signals to responses respectively. While the former network from cues to intracellular signals operate through signal transduction mechanisms, such as protein phosphorylation and involve significant cross-talk between different pathways with many layers of interacting proteins, the network from signals to responses often include transcriptional events with much smaller number of components. Our results suggest that when using one-to-one mapping between signals and responses both types of cascades tend to operate though non-linear interactions, although mappings from cues to intracellular signals are likely to be more dramatically nonlinear than mappings from signals to responses. In part, this agrees with previous studies on apoptosis [5] and T-cell activation networks (Kemp et al., Signal Combinations Downstream of T Cell Receptor Activation Predict IL-2 Response, submitted), where PLSR accurately predicted cellular responses from signals using linear functions, although failed to predict signaling from cues to intracellular signals. The significant difference of those approaches from ours is that both used multiple time slices of the signaling network to predict cellular responses. An approximately multi-linear relationship between signals and responses, when multiple time slices of the signaling network are taken into account, might have important applications for predictive modeling of cell decision processes in clinical applications, considering that linear methods have much higher reproducibility and robustness. It also highlights the importance of choosing the optimal number of time points when producing cues – signals – responses data for predictive and descriptive modeling of signaling networks, as was described in the introduction.

Our study could be extended in several ways. First, better approaches for analysis of Isomap projections could be used. Second, other non-linear dimensionality reduction techniques could be applied to study signaling networks in addition to Isomap as used here. A disadvantage of Isomap is the requirement for dense coverage of manifold with datapoints, which is necessary for accurate approximation of submanifold with geodesic distance. Through sampling we have shown that the data in the Cytokine compendium is sufficiently dense for Isomap to approximate nonlinear submanifold. However, this may not be true for other datasets. Research on spectral methods for dimensionality reduction continues at a rapid pace. Other algorithms closely related to the Isomap and LLE include hessian eigenmaps [23], local tangent space alignment [24], Laplacian eigenmaps [25] and locally linear isomaps [26]. These methods allow handling of manifolds with more complex geometries that are more robust to noise and outliers and have the ability to scale to larger data sets. Application of these techniques may provide further advances in systems wide analysis of signaling networks.

## Conclusion

The construction of predictive and descriptive models for the analysis of signaling networks is essential for understanding cell decision processes at the systems wide level. Cues – signals – responses paradigm provides a unifying framework for data analysis and modeling of signaling networks. The comparison of different treatment conditions in terms of changes in the intracellular signaling molecules they induce is particularly important. Here we developed the extended Isomap algorithm for the non-linear dimensionality reduction and applied it to the analysis of the two signaling networks: apoptosis signaling networks induced by treatment with TNF, EGF and Insulin in cancer cells and multiple signaling pathways derived from AfCS double ligand screen dataset. We show that Extended Isomap can find clusters corresponding to different treatment conditions through the non-linear dimensionality reduction of the space of intracellular signaling molecules. By applying neural networks sensitivity analysis we also recovered the subset of signaling proteins that best explain Isomap projection from the original into the low dimensional subspace. Finally, we demonstrated how Isomap components can be used in a supervised learning content for predictive modeling of apoptosis intensity. We conclude that extended Isomap approach can be used for visualization, predictive and descriptive modeling of signaling networks.

## Methods

### Cytokine compendium data normalisation and transformation

Data in the Cytokine compendium used in the study were normalized for different assays and reproducibility between time course experiments done at different times. All the measurements were normalized to the 0 time point. To scale the data for estimation of Isomap geodesic distances appropriately, each molecular signal at each individual time point (e.g. TNF 100 ng treatment at 5 min) was normalized to unit variance. For neural networks application the data was scaled into [0 1] range.

### Extended Isomap approach

#### Original Isomap and determination of k nearest neighbors

The Isomap algorithm attempts to find a low-dimensional representation of a high dimensional data set that most faithfully preserves pairwise distances between input patterns measured along the submanifold from which they were sampled. Isomap achieves this in three distinct steps. In the first step, Isomap finds k-nearest neighbors from the distance matrix for each input data point in *n *dimensions and constructs a sparse k-nearest neighbors graph where each data point is connected to its k-nearest neighbors. The edges are then assigned weights based on the Euclidean distance between nearest neighbors. The second step estimates the pairwise distances d_ij _between all data points *(i, j) *by finding shortest paths through the k-nearest neighbors graph using Djikstra's algorithm [27]. Finally, in the third step, the pairwise instances d_ij, _estimated in the previous step, are fed as input into Classical Metric MDS. The MDS yields a low-dimensional representation ψ_j _∈ *R*_m _for which (ψ_i _- ψ_j_)^2 ^≈ *d*_ij2_. The value of m required for a faithful low-dimensional representation is estimated by the number of significant eigenvalues in the Gram matrix constructed by MDS.

The critical parameter that determines Isomap performance is the number of nearest neighbors, k. If *k *is too small, Isomap cannot capture enough information on local dimensionality, which leads to appearance of disconnected components and discrete points in the low-dimensional space. If k is too large, neighborhoods considered by the algorithm are more global than local and Isomap may fail to find a low-dimensional representation of the network. Consequently, the optimal value for k was determined on the basis of stability of the low-dimensional representation. To assess the stability for each value of k, 5% of the data was randomly removed from the Cytokine compendium and presence/absence of disconnected components as well as number of significant eigenvalues was recorded for 20 iterations. Based on this data the value of k = 3 was selected as the optimal number of nearest neighbors.

#### Graph-based clustering in the Isomap components

Given a collection of *n *datapoints (treatment conditions) *S*, the similarity graph *G*_*s *_is obtained by modeling each treatment as a vertex and having an edge between each pair of vertices whose weight is equal to the similarity between the corresponding treatments in the signaling space. A MinMaxCut criterion function was used to measure the overall clustering quality and find the optimal solution. It is defined as

∑r=1kcut(Sr,S−Sr)∑di,dj∈Srsim(di,dj)
 MathType@MTEF@5@5@+=feaafiart1ev1aaatCvAUfKttLearuWrP9MDH5MBPbIqV92AaeXatLxBI9gBaebbnrfifHhDYfgasaacH8akY=wiFfYdH8Gipec8Eeeu0xXdbba9frFj0=OqFfea0dXdd9vqai=hGuQ8kuc9pgc9s8qqaq=dirpe0xb9q8qiLsFr0=vr0=vr0dc8meaabaqaciaacaGaaeqabaqabeGadaaakeaadaaeWbqaamaalaaabaGaem4yamMaemyDauNaemiDaqNaeiikaGIaem4uam1aaSbaaSqaaiabdkhaYbqabaGccqGGSaalcqWGtbWucqGHsislcqWGtbWudaWgaaWcbaGaemOCaihabeaakiabcMcaPaqaamaaqababaGaem4CamNaemyAaKMaemyBa0MaeiikaGIaemizaq2aaSbaaSqaaiabdMgaPbqabaGccqGGSaalcqWGKbazdaWgaaWcbaGaemOAaOgabeaakiabcMcaPaWcbaGaemizaq2aaSbaaWqaaiabdMgaPbqabaWccqGGSaalcqWGKbazdaWgaaadbaGaemOAaOgabeaaliabgIGiolabdofatnaaBaaameaacqWGYbGCaeqaaaWcbeqdcqGHris5aaaaaSqaaiabdkhaYjabg2da9iabigdaXaqaaiabdUgaRbqdcqGHris5aaaa@5BAF@

where cut *(S*_*r*_, *S*-*S*_*r*_*) *is the edge-cut between the vertices in *S*_*r *_to the rest of the vertices in the graph *S*-*S*_*r *_. The edge-cut between two sets of vertices *A *and *B *is defined to be the sum of the edges connecting vertices in *A *to vertices in *B*. The motivation behind this criterion function is that the clustering process can be viewed as that of partitioning the treatments into groups by minimizing the edge-cut of each partition. However, for many problems this criterion function has trivial solutions that can be achieved by assigning to the first *k *- 1 clusters a single data point that shares very few terms with the rest, and then assigning the rest to the *k*th cluster. For this reason each edge-cut is scaled by the sum of the internal edges, which leads to the balanced clustering solutions.

Graph-based clustering itself is executed in three stages: first, a distance matrix between each data point in low-dimensional subspace is computed using Euclidian distances, followed by a sparsification step where only 20 k-nearest neighbors of each datapoint are retained. Finally, the resulting sparse graph is partitioned into clusters using a multilevel k-way partitioning scheme for irregular graphs [28]. The advantage of this approach is that it allows detection of irregular graphs of different densities and sizes.

#### Interpretation of Isomap projections by neural networks

Different methods have been proposed for interpreting what has been learned by a feed-forward neural network. These interpretative methods can be divided in two types of methodologies: analysis based on the magnitude of weights and sensitivity analysis. Analysis based on the magnitude of weights groups together procedures that are based exclusively on the values stored in the static matrix of weights to determine the relative influence of each input variable on each one of the network outputs. In contrast, sensitivity analysis is based on the measurement of the effect that is observed in the output y_k _due to the change that is produced in the input x_i_. We used a form of sensitivity analysis based on the "missing value problem". One way to carry out this type of analysis, called clamping technique [29], consists of comparing the error made by the network from the original patterns with the error made when restricting the input of interest to a fixed value (in general the average value) for all patterns. However, we adopted a more sophisticated approached based on data imputation, where one attempts to predict the unknown input variables conditioned upon those which are known, by constructing auxiliary models – the imputed values are used to fill in the gaps before the main model is used to predict the output [30]. We analyzed sensitivity by replacing each variable in turn with missing values, and assessing the effect upon the output error produced by the neural network.To define the sensitivity of a particular variable, *V*, we first run the network on a set of test cases, and accumulate the network error *E*. We then run the network again using the same cases, but this time replacing the observed values of *V *with values estimated by the missing value procedure. Next we estimate the corresponding network error *E(V) *and rank variables by the ratio *E(V)/E*, where variables with high ratio contribute most to the mapping function of neural networks.

In our application, three-layered perceptron networks are used. The activation functions of the hidden layer and the output layer are selected as logistic function and pure linear function respectively. A two-stage process is used to train the network: 30 epochs of scaled conjugate gradient descent followed by Quasi-Newton BFGS optimisation for 100 epochs. This approach gives improved performance due to greater stability in the error function Hessian matrix once the gradient-descent has located a reasonable starting point for the second-order descent [31]. We also scaled the input variables into a consistent range [0, 1] using the Minimax approach. The number of hidden nodes was chosen moderately small to ensure the generalization (4–5). The network was trained 100 times with random initialization. The selection subset comprising 25% of the normalized dataset was chosen first without bootstrapping (i.e. the selection subset is sampled without replacement); then, the training subset is bootstrapped from the other 75% of the data and the test set includes remaining variables not sampled by the bootstrap.

#### Choice of class labels

The results produced by Isomap were compared with those obtained by PCA. To compare the two algorithms in an unbiased way we have used both exploratory analysis and a supervised classification approach. To perform classification it was first necessary to select class labels (apoptosis intensity) for each time course treatment, for instance activity of signaling network five minutes after stimulation with 100 ng of TNF. Unfortunately, we could not use the results of apoptosis assays in the Cytokine compendium directly as they were measured only twice during the 24 -hour time course (with a third measurement made at 48 hours after stimulation). With only two apoptosis measurements for 13 measurements of signaling network activity, the entire time course of distinct cytokine treatments (e.g. TNF 100 ng) corresponds to a single measurement of apoptosis intensity (e.g. high apoptosis intensity). This assumption was checked against compendium dataset to insure consistency, and it allowed us to consider the 12 apoptosis measurements for each treatment condition as signatures for class labels in classification. Expectation Maximization (EM) clustering was used to discretize continuous apoptosis measurement data into three different classes which represent strong, medium or absence of apoptotic response.

#### Classifiers comparison

Having derived class labels we used SVM (1-norm soft-approximation, capacity = 10000, radial bases function kernel with variance 0.2), k-nearest neighbors, k-NN (k = 3, number of iterations for 10-fold cross-validation – 1000 to break neighboring ties) and quadratic discriminant analysis (QDA) to compare the 3-dimensional signaling network reconstructions found by PCA, Isomap and the full model comprising all 19 molecular signals (dimensions). Optimal SVM parameters were estimated using a cross validation loop. For comparing hypotheses produced by PCA, Isomap or original dataset with three different algorithms, we used 10-fold cross validation. 95% confidence intervals for the error rates were estimated using binomial test. ANOVA was used to estimate statistical significance of differences between error rates reported for each of the classifiers and the Tukey-Kramer test was used to perform post hoc comparison of the means. For testing k-NN for classification accuracy of apoptosis at late time points, we withheld late time points for each of 10 different cytokine treatment conditions and used it in the test set, starting with one condition (24 hours), and until 50% of compendium data was withheld (4 to 24 hours).

#### PLS Discriminant Analysis

Computational analysis was done using Statistca (StatSoft, Inc.) as described in details elsewhere [4]. The software uses the non-linear iterative partial least-squares algorithm (NIPALS) to perform decompositions and regressions. Goodness of prediction Q2 was evaluated using 10 fold-cross validation. Briefly, cross validation is performed by omitting 10% of observations (13 data points) from the model development and then using the model to predict the Y-block values for the removed observations.

#### Choice of Principal components (PC) using cross validation

We have used cross validation to find and estimate the number of PC as follows: one entry of the Cytokine compendium data matrix are kept out of the PCA model development, and then predicted by the model, and compared with the actual values. The prediction error sum of squares (PRESS) is the squared differences between observed and predicted values for the data kept out of the model fitting was calculated according to the formula

PRESS(m)=∑i=1n∑j=1p(mX^ij−Xij)2
 MathType@MTEF@5@5@+=feaafiart1ev1aaatCvAUfKttLearuWrP9MDH5MBPbIqV92AaeXatLxBI9gBamXvP5wqSXMqHnxAJn0BKvguHDwzZbqegyvzYrwyUfgarqqtubsr4rNCHbGeaGqiA8vkIkVAFgIELiFeLkFeLk=iY=Hhbbf9v8qqaqFr0xc9pk0xbba9q8WqFfeaY=biLkVcLq=JHqVepeea0=as0db9vqpepesP0xe9Fve9Fve9GapdbaqaaeGacaGaaiaabeqaamqadiabaaGcbaGaemiuaaLaemOuaiLaemyrauKaem4uamLaem4uamLaeiikaGIaemyBa0MaeiykaKIaeyypa0ZaaabCaeaadaaeWbqaaiabcIcaOmaaBaaaleaacqWGTbqBaeqaaOGafmiwaGLbaKaadaWgaaWcbaGaemyAaKMaemOAaOgabeaakiabgkHiTiabdIfaynaaBaaaleaacqWGPbqAcqWGQbGAaeqaaOGaeiykaKYaaWbaaSqabeaacqaIYaGmaaaabaGaemOAaOMaeyypa0JaeGymaedabaGaemiCaahaniabggHiLdaaleaacqWGPbqAcqGH9aqpcqaIXaqmaeaacqWGUbGBa0GaeyyeIuoaaaa@626D@

where *m *is the current number of PC and _*m *_X^
 MathType@MTEF@5@5@+=feaafiart1ev1aaatCvAUfKttLearuWrP9MDH5MBPbIqV92AaeXatLxBI9gBaebbnrfifHhDYfgasaacH8akY=wiFfYdH8Gipec8Eeeu0xXdbba9frFj0=OqFfea0dXdd9vqai=hGuQ8kuc9pgc9s8qqaq=dirpe0xb9q8qiLsFr0=vr0=vr0dc8meaabaqaciaacaGaaeqabaqabeGadaaakeaacuWGybawgaqcaaaa@2DF5@_*ij *_is the predicted value of *X*_*ij*_. We have used Krzanowski cross-validation approach where each entry is removed from the data and PCs were constructed by the NIPALS algorithm using Statistca (StatSoft, Inc.).

## Authors' contributions

SI conceived of and designed the study, carried out the data analysis and visualization, developed the Matlab scripts for data manipulation, and drafted the manuscript. JDA contributed to the design of the study, interpretation of results and editing of the manuscript. All authors read and approved the final manuscript.

## Supplementary Material

Additional file 1 PCA projections of the Cytokine compendium and AfCS double ligand screen datasets with supplementary tables. Provides figures of PCA projections of the Cytokine compendium and AfCS double ligand screen datasets. List of measured protein signals and apoptosis markers used in the construction of the Cytokine compendium. Mean ranking of the molecular signals in the Cytokine compendium dataset obtained based on the sensitivity analysis with neural networks. List of ligands used in the AfCS double ligand screen with functional coherence scores of the clusters found using Isomap and PCA. Click here for file

Additional file 2 The guide to the software used in the paper. provides brief description of the software that was used in the manuscript, software availability additional justification for particular parameters used Click here for file

Additional file 3 Script to generate tab delimited file from raw AfCS double ligand screen dataset. Example perl script to generate tab delimited file from raw AfCS double ligand screen dataset. Click here for file

Additional file 4 Scripts for pre-processing AfCS double ligand screen dataset. Example perl script to pre-process tab delimited file of AfCS double ligand screen dataset. Click here for file

Additional file 5 Matlab script to perform classification with SVM. Example matlab script to perform classification with SVM (10 fold cross validation) in the Isomap first two components. Click here for file
