# Supplementary material for: Non-linear dimensionality reduction of signaling networks
Source: BMC Syst Biol. 2007 Jun 8;1:27. doi: 10.1186/1752-0509-1-27 (PMC1925119; doi:10.1186/1752-0509-1-27)
Supplement: Additional file 1 — PCA projections of the Cytokine compendium and AfCS double ligand screen datasets with supplementary tables. Provides figures of PCA projections of the Cytokine compendium and AfCS double ligand screen datasets. List of measured protein signals and apoptosis markers used in the construction of the Cytokine compendium. Mean ranking of the molecular signals in the Cytokine compendium dataset obtained based on the sensitivity analysis with neural networks. List of ligands used in the AfCS double ligand screen with functional coherence scores of the clusters found using Isomap and PCA. [file 1752-0509-1-27-S1.doc]

**Supplementary Information**

**Supplementary Figure 1** **PCA projection of the Cytokine compendium dataset**

The cytokine compendium was projected into the two-dimensional space of the first two principal components with largest eigenvalues. For comparison, the assignment of data points to six clusters is the same as in Figure 4 for Isomap.

**Supplementary Figure 2 PCA projection of the AfCS double ligand screen dataset**

The cytokine compendium was projected into the two-dimensional space of the first two principal components with largest eigenvalues. Functional coherence of the clusters is listed in Supplementary Table 4b.

**Supplementary Table 1** List of measured protein signals and apoptosis markers used in the construction of the Cytokine compendium. Also indicated experimental assays used for measuring each signal (reproduced with permission from ref 5 in the main text).

| **Protein name** | **Biological function** | **Molecular signal** | **Assay** |
| --- | --- | --- | --- |
| IKK | Ser kinase | Kinase activity | Kinase assay |
| JNK1 | Ser-Thr kinase | Kinase activity | Kinase assay |
| MK2 | Ser-Thr kinase | Kinase activity | Kinase assay |
| EGFR | Receptor Tyr kinase | Phosphorylation (Tyr^1068^) | Ab microarray |
|  |  | Total amount | Ab microarray |
|  |  | Phospho/total ratio | Ab microarray |
| MEK | Dual-specificity kinase | Phosphorylation (Ser^217^/Ser^221^) | Western blot |
| ERK | Ser-Thr kinase | Kinase activity | Kinase assay |
| IRS1 | Adaptor-scaffold | Phosphorylation (Ser^636^) | Western blot |
|  |  | Phosphorylation (Tyr^896^) | Western blot |
| Akt | Ser-Thr kinase | Phosphorylation (Ser^473^) | Ab microarray |
|  |  | Total amount | Ab microarray |
|  |  | Kinase activity | Kinase assay |
|  |  | Phosphorylation (Ser^473^) | Western blot |
|  |  | Phospho/total ratio | Ab microarray |
| FKHR | Transcription factor | Phosphorylation (Ser^256^) | Western blot |
| Caspase-8 | Cys protease | Zymogen amount | Western blot |
|  |  | Cleaved amount | Western blot |
| Caspase-3 | Cys protease | Zymogen amount | Western blot |

### Supplementary Table 2 Mean ranking of the molecular signals in the Cytokine compendium dataset. The ranking was obtained based on the sensitivity analysis with neural networks

|  | **Mean** | **Std.Dev.** |
| --- | --- | --- |
| pAkt(AA) | 4,33333 | 3,278719 |
| pIRS1-636 | 5,33333 | 5,500000 |
| pMEK | 5,77778 | 2,728451 |
| MK2 | 6,22222 | 4,684490 |
| ProC3 | 6,22222 | 5,932491 |
| ERk | 7,00000 | 2,598076 |
| ClvC8 | 7,00000 | 5,172040 |
| JNK | 9,22222 | 3,345810 |
| ptAkt | 10,22222 | 6,869821 |
| ProC8 | 10,33333 | 4,873397 |
| pEGFR | 10,88889 | 4,539946 |
| tEGFR | 11,11111 | 4,728754 |
| Akt | 12,33333 | 4,242641 |
| pIRS1-896 | 12,44444 | 4,390647 |
| pFKHR | 12,77778 | 3,800585 |
| pAkt(IB) | 13,22222 | 4,409586 |
| ptEGFR | 14,11111 | 3,919325 |
| IKK | 15,55556 | 4,475241 |
| tAkt | 15,88889 | 2,891559 |

**Supplementary Table 3** List of ligands used in the AfCS double ligand screen with abbreviations, alternate names and concentration

| **Abbreviation** | **Ligand (with alternate names) and concentration** |
| --- | --- |
| 2MA | 2-Methylthioadenosine 5'-triphosphate tetrasodium ; 2-methyl-thio-ATP; 500μM |
| 848 | Resiquimod (R-848); 100nM |
| C5A | Complement C5a, recombinant human; 100nM |
| GMF | Granulocyte-macrophage colony-stimulating factor, recombinant mouse, G-MCSF, 10pM. |
| IL-4 | Interleukin-4, I04, IL4, recombinant mouse, 250pM |
| IL-6 | Interleukin-6, I06, IL6, recombinant mouse, 300pM |
| IL-10 | Interleukin 10, I10, IL10, recombinant human, 350pM |
| IL1β | Interleukin-1b, I1B, IL1b, recombinant mouse, 20pM |
| IFα | Interferon alpha, IFA, IFNa, recombinant mouse, 1nM |
| IFβ | Interferon beta, IFB, IFNb, recombinant mouse, 100pM |
| IFγ | Interferon gamma, IFG, IFNg, recombinant mouse, 300pM |
| ISO | Isoproterenol; isoprenaline, isopropylnoradrenaline , isopropterenol hydrochloride, 50nM |
| LPA | Lysophosphatidic acid, 1-oleoyl-2-hydroxy-sn-glycero-3-phosphate; 2.5μM |
| LPS | Lipopolysaccharide; 100ng/ml, with added LPS-binding protein (LBP) 250pM |
| MCF | Macrophage colony-stimulating factor, recombinant mouse; M-CSF; 200pM |
| P2C | Pam2Cys-SKKKK x 3 TFA; S-[2,3-bis(palmitoyloxy)-(2RS)-propyl]-[R]-cysteinyl-[S]-seryl-[S]-lysyl-[S]-lysyl-[S]-lysyl-[S]-lysine x 3 CF3COOH, PAM 2; 350nM |
| P3C | Pam3Cys-SKKKK x 3 HCl; (N-Palmitoyl-S-[2,3-bis(palmitoyloxy)-(2RS)-propyl]-[R]-cysteinyl-[S]-seryl-[S]-lysyl-[S]-lysyl-[S]-lysyl-[S]-lysine x 3 HCl), PAM 3; 1μM |
| PAF | Platelet activating factor, L-alpha-phosphatidylcholine, beta-acetyl-gamma-O-alkyl, 100nM |
| S1P | Sphingosine-1-phosphate; 1μM |
| TGFβ | Transforming growth factor-beta 1, recombinant human, Chinese hamster ovary cells; TGF-β1; TGF; TGFb; 10pM |
| UDP | Uridine 5'-diphosphate trisodium salt dihydrate; 25μM |

**Supplementary Table 4** Functional coherence scores of the clusters found using Isomap and PCA in the AfCS double ligand screen dataset.

| ligands/clusters | GMF | C5A | I06 | IFA | LPS | PAF | P3C | 2MA | I04 | MCF |
| --- | --- | --- | --- | --- | --- | --- | --- | --- | --- | --- |
| Isomap |  |  |  |  |  |  |  |  |  |  |
| 1 | 0,2200644 | 0,1452986 | 0,2283841 | 0,3144975 | 0,1824241 | 0,2158095 | 0,1280777 | 0,2114981 | 0,2134273 | 0,1681677 |
| 2 | 0,3130266 | 0,2499734 | 0,3107364 | 0,2100606 | 0,101538 | 0,0800803 | 0,199608 | 0,1618659 | 0,1484931 | 0,201606 |
| 3 | 0,2071883 | 0,1119249 | 0,1552931 | 0,1421261 | 0,3435007 | 0,1219094 | 0,313518 | 0,1368972 | 0,1507047 | 0,2314026 |
| 4 | 0,1501528 | 0,3053696 | 0,210828 | 0,1454134 | 0,2071019 | 0,2598523 | 0,1387945 | 0,1145974 | 0,3180176 | 0,2056028 |
| 5 | 0,1095679 | 0,1874335 | 0,0947585 | 0,1879023 | 0,1654353 | 0,3223484 | 0,2200019 | 0,3751414 | 0,1693573 | 0,1932209 |
| PCA |  |  |  |  |  |  |  |  |  |  |
| 1 | 0,2139497 | 0,1498692 | 0,04972 | 0,1925585 | 0,3575347 | 0,1558119 | 0,2491442 | 0,1466052 | 0,1681921 | 0,1807243 |
| 2 | 0,2300534 | 0,0805748 | 0,2940427 | 0,2070522 | 0,192223 | 0,0837698 | 0,3297194 | 0,2101867 | 0,1291798 | 0,2186181 |
| 3 | 0,1165303 | 0,3795707 | 0,2680978 | 0,1415872 | 0,136315 | 0,2927837 | 0,112735 | 0,0838428 | 0,3297884 | 0,2436234 |
| 4 | 0,1184055 | 0,1818336 | 0,095249 | 0,1752209 | 0,1826486 | 0,3183895 | 0,198258 | 0,355747 | 0,1657065 | 0,1557968 |
| 5 | 0,3210611 | 0,2081517 | 0,2928905 | 0,2835812 | 0,1312787 | 0,1492451 | 0,1101433 | 0,2036183 | 0,2071331 | 0,2012375 |
|  |  |  |  |  |  |  |  |  |  |  |
|  |  |  |  |  |  |  |  |  |  |  |
| ligands/clusters | I1B | IFG | IFB | ISO | TGF | UDP | LPA | 848 | I10 | P2C |
| Isomap |  |  |  |  |  |  |  |  |  |  |
| 1 | 0,2554271 | 0,3714949 | 0,362683 | 0,1564331 | 0,1625643 | 0,1314367 | 0,1355718 | 0,178585 | 0,2735921 | 0,1215203 |
| 2 | 0,2274747 | 0,1792054 | 0,111377 | 0,2031663 | 0,1809678 | 0,2048428 | 0,1207356 | 0,2982032 | 0,3045648 | 0,0450924 |
| 3 | 0,132265 | 0,1865379 | 0,2590403 | 0,2086469 | 0,2805963 | 0,197994 | 0,216987 | 0,120097 | 0,1756255 | 0,3559425 |
| 4 | 0,184533 | 0,1610317 | 0,1174572 | 0,1883583 | 0,2544629 | 0,1614257 | 0,2938311 | 0,1935278 | 0,1347657 | 0,1950938 |
| 5 | 0,2003001 | 0,1017301 | 0,1494425 | 0,2433954 | 0,1214088 | 0,3043009 | 0,2328746 | 0,209587 | 0,1114518 | 0,282351 |
| PCA |  |  |  |  |  |  |  |  |  |  |
| 1 | 0,1626358 | 0,2222095 | 0,1510295 | 0,1400003 | 0,2993914 | 0,2440824 | 0,2395685 | 0,166889 | 0,0221843 | 0,4038028 |
| 2 | 0,2248421 | 0,1592899 | 0,3479943 | 0,2508967 | 0,1238178 | 0,0787362 | 0,1639276 | 0,1794505 | 0,4770816 | 0,1447322 |
| 3 | 0,2163922 | 0,1573379 | 0,1269156 | 0,1486932 | 0,2370746 | 0,1675075 | 0,266899 | 0,2220511 | 0,1522452 | 0,169665 |
| 4 | 0,1691341 | 0,1418962 | 0,1570641 | 0,2592599 | 0,1676521 | 0,3273135 | 0,1919218 | 0,2192302 | 0,093497 | 0,1989176 |
| 5 | 0,2269958 | 0,3192665 | 0,2169965 | 0,2011499 | 0,172064 | 0,1823604 | 0,1376831 | 0,2123792 | 0,2549919 | 0,0828823 |

### Supplementary Table 5 Mean ranking of the molecular signals in AfCS double ligand screen dataset. The ranking was obtained based on the sensitivity analysis with neural networks

|  | **Mean** | **Std.Dev.** |
| --- | --- | --- |
| AKT | 6,22222 | 1,787301 |
| ERK1 | 16,66667 | 3,427827 |
| ERK2 | 14,33333 | 6,284903 |
| EZR | 6,00000 | 1,936492 |
| GSKA | 10,44444 | 3,045944 |
| GSKB | 16,00000 | 5,722762 |
| JNKL | 4,66667 | 1,000000 |
| JNKS | 1,66667 | 2,000000 |
| MOE | 15,88889 | 3,855011 |
| P38 | 5,55556 | 4,719934 |
| P40 | 17,00000 | 2,738613 |
| P65 | 13,22222 | 2,223611 |
| PKCD | 18,44444 | 3,395258 |
| PKCM | 15,33333 | 2,061553 |
| RSK | 16,11111 | 4,807402 |
| S6 | 9,33333 | 2,345208 |
| SMD2 | 13,55556 | 2,006932 |
| ST1A | 9,00000 | 2,692582 |
| ST1B | 11,11111 | 3,480102 |
| ST3 | 4,11111 | 6,333333 |
| ST5 | 6,33333 | 4,183300 |
