# Supplementary material for: Non-linear dimensionality reduction of signaling networks
Source: BMC Syst Biol. 2007 Jun 8;1:27. doi: 10.1186/1752-0509-1-27 (PMC1925119; doi:10.1186/1752-0509-1-27)
Supplement: Additional file 2 — The guide to the software used in the paper. provides brief description of the software that was used in the manuscript, software availability additional justification for particular parameters used [file 1752-0509-1-27-S2.doc]

**The guide to the software supplement**

Extended Isomap approach is based on the use of the following software: Matlab Bioinformatics toolbox (The MathWorks, Inc); Statistca (StatSoft, Inc.) and CLUTO 2.1.2 clustering software. In addition custom written matlab and perl script are listed below.

1) CLUTO 2.1.2

CLUTO is a software package for clustering low- and high-dimensional datasets and for analyzing the characteristics of the various clusters. CLUTO is well-suited for clustering data sets arising in many diverse application areas including information retrieval, customer purchasing transactions, web, GIS, science, and biology.

CLUTO's distribution consists of both stand-alone programs and a library via which an application program can access directly the various clustering and analysis algorithms implemented in CLUTO. The common parameters for executing CLUTO stand-alone version in the Extended Isomap are given below

*vcluster datafile.mat 4 –grmodel=sl -clmethod=graph - nnbrs =20 where 4 number of clusters, clmethod – clustering methods, nnbrs – number of nearest neighbors*

CLUTO 2.1.2 that can be downloaded from <http://glaros.dtc.umn.edu/gkhome/cluto/cluto/overview>

2) Example scripts

1. svmclass.m - Example script to perform classification with SVM (10 fold cross validation) in the Isomap first two components. DATA2 is a data file of the cytokine compendium
2. AfCS_proc_scrip1.pl - Takes as input AfCS double ligand screen data (wesrrwo.txt – file generated by concatenation of individual files from western_double_RAW264.7.tar.gz downloaded from AfCS site [www.signaling-gateway.org/](http://www.signaling-gateway.org/) ) and generates tab delimited file (perlcountlines2.txt) with 4 columns as follows: ligand, phosphoprotein, time, and fold changes
3. AfCS_proc_scrip2.pl - Takes as input tab delimited file (perlcountlines2.txt) with 4 columns - ligand, phosphoprotein, time, and fold changes of AfCS double ligand screen data; tab delimited file (perlcountlines7.txt) of ligands names in double screen data and produces matrix of ligand treatments (for 3 time points) and corresponding phoshoprotein measurements.
